# Comments: Myositis-specific and myositis-associated autoantibody profiles and their clinical associations in a large series of patients with polymyositis and dermatomyositis

**DOI:** 10.1016/j.clinsp.2022.100077

**Published:** 2022-07-23

**Authors:** Bruno Silva de Araujo Ferreira, Bernardo Matos da Cunha, Larissa Aniceto Moreira, Michel Fabrício Silveiro e Fonseca, Eduardo Boiteux Uchôa Cavalcante

**Affiliations:** a Centro Universitário Serra dos Órgãos (UNIFESO), Teresopolis, RJ, Brazil; b Centro Universitário do Planalto Central Apparecido dos Santos (UNICEPLAC), Brasília, DF, Brazil; c Rede SARAH de Hospitais de Reabilitação, Brasília, DF, Brazil

Dear Editor,

I have read the article “Myositis-specific and myositis-associated autoantibody profiles and their clinical associations in a large series of patients with polymyositis and dermatomyositis” by Cruellas et al.^1^ with great interest because it is the only published study investigating Brazilian myositis patients' antibody profiles.

Idiopathic Inflammatory Myopathies (IIMs) are a group of autoimmune diseases that mainly affect the muscles. Other organs may also be affected, such as the skin, lungs, gastrointestinal tract, and heart. In addition to the possible effects on these organs, there is also an increased risk for cancer.

Myositis antibodies are important for diagnosis and prognosis since some antibodies may predict organ involvement and response to therapy. Furthermore, some of the antibodies are myositis-specific, so they could be a useful investigating tool. Indeed, current classification criteria have added the anti-Jo1 antibody, as it is commercially available in most health services. Many countries have studied antibody prevalence, and their frequencies range from 60% to 81%.^2^, ^3^, ^4^, ^5^, ^6^

Myositis antibody prevalence in the Cruellas et al. cohort was lower than that reported in the literature. The authors justified that it might have been due to the long period between blood collection and analysis (approximately 10 years). As plasma is a biological product, it may have degraded by the time the authors tested it, even if the samples were stored at -80°C (ultra-low temperature freezers).^7^^,^^8^ Furthermore, some antibodies were not tested.

In our study, we tested our patients with the *Euroimmun* kit, as Cruellas et al. did, but we analyzed the samples after a recent blood collection. Moreover, our kit included four additional autoantibodies. We found a higher prevalence of antibody-positive patients than in the previously reported study (80% × 54.1%). Regarding Myositis-Specific Antibodies (MSAs) and Myositis-Associated Antibodies (MAAs), we found positivity rates of 66% and 36%, respectively, which contrasted with the rates of 34% and 41% in the former study (Table 1). Four patients (8%) had more than one MSA. It is noteworthy that we found higher frequencies of anti-Mi2 and anti-SRP, probably due to selection bias since the rehabilitative nature of our hospital would gather patients with more significant muscular involvement.^4^^,^^9^^,^^10^

**Table 1:** Comparison between myositis-patient cohorts regarding myositis-specific antibodies and myositis-associated antibodies profiles.

	**Antibody**	**Sarah(n = 50)**	**USP(n = 222)****^1^**	**Literature****^2^****^-^****^6^**
**MSA**	Jo1	10%	18.9%	10%‒20%
	Mi2	22%	8.1%	5%‒10%
	SRP	18%	3.2%	4%‒13%
	PL7	4%	2.7%	<5%
	PL12	0%	3.2%	<5%
	EJ	0%	2.7%	<2%
	OJ	0%	0%	<2%
	TIF1y	8%	NA	7%‒30%
	MDA5	6%	NA	1%‒30%
	NXP2	6%	NA	2%‒20%
	SAE1	2%	NA	1%‒3%
**MAA**	Ku	10%	4.1%	4%‒30%
	PM/Scl100	12%	2.3%	5%‒8%
	PM/Scl75	6%	2.7%	
	Ro52	20%	36.9%	6%‒25%
**More than 1 MSA**		8%		0.2%‒15.6%
**Positivity**		80.0%	54.1%	60%‒81%

USP, Universidade de São Paulo; MSA, Myositis Specific Antibodies; MAA, Myositis Associated Antibodies; Jo-1, anti-histidyl-tRNA synthetase; SRP, anti-Signal Recognition particle; Mi-2, anti-Mi2; PL-7, anti-threonyl-tRNA synthetase; PL-12, anti-alanyl-tRNA synthetase; EJ, anti-glycol-tRNA synthetase; OJ, anti-isoleucyl-tRNA synthetase; TIF1 γ, anti-Transcription Intermediary Factor 1γ; MDA5, anti-Melanoma Differention Associated Protein 5; NXP2, anti-Nuclear Matrix Protein 2; SAE1, anti-Small ubiquitin-like modifier Activating Enzyme; Ku, anti-Ku; PM/Scl, anti-PM/Scl; Ro52, anti-Ro52.

Some limitations in our study are noteworthy. First, our hospital is a rehabilitation centre, and there could possibly be a selection bias towards more severe cases. Second, we did not use immunoprecipitation to confirm our results. Finally, we did not test seven patients whose diagnoses had been performed in other services, but we believe it did not introduce selection bias.

Our data show that the frequency of myositis-specific and myositis-associated autoantibodies is higher than that first reported in Brazilian myositis patients. This would encourage the use of these tests more often when IIM is suspected. Therefore, we recommend that a myositis antibody panel be generally available. Rheumatologists could classify patients faster and begin treatment earlier.

## Conflicts of interest

The authors declare no conflicts of interest.
